# Formation and metabolism of 6-(1-acetol)-8-(1-acetol)-rutin in foods and *in vivo*, and their cytotoxicity

**DOI:** 10.3389/fnut.2022.973048

**Published:** 2022-08-02

**Authors:** Min Chen, Pengzhan Liu, Hua Zhou, Caihuan Huang, Weiye Zhai, Yuantao Xiao, Juanying Ou, Jun He, Hani El-Nezami, Jie Zheng

**Affiliations:** ^1^Department of Food Science and Engineering, Jinan University, Guangzhou, China; ^2^School of Food Science and Engineering, South China University of Technology, Guangzhou, China; ^3^Dongguan Silang Foods Co., Ltd., Dongguan, China; ^4^Institute of Food Safety and Nutrition, Jinan University, Guangzhou, China; ^5^Institute of Laboratory Animal Science, Jinan University, Guangzhou, China; ^6^School of Biological Sciences, University of Hong Kong, Pok Fu Lam, Hong Kong SAR, China; ^7^School of Medicine, Institute of Public Health and Clinical Nutrition, University of Eastern Finland, Kuopio, Finland; ^8^Guangdong-Hong Kong Joint Innovation Platform for the Safety of Bakery Products, Guangzhou, China

**Keywords:** rutin, methylglyoxal (MGO), adducts, food, absorption, accumulation, *in vivo*

## Abstract

Methylglyoxal (MGO) is a highly reactive precursor which forms advanced glycation end-products (AGEs) *in vivo*, which lead to metabolic syndrome and chronic diseases. It is also a precursor of various carcinogens, including acrylamide and methylimidazole, in thermally processed foods. Rutin could efficiently scavenge MGO by the formation of various adducts. However, the metabolism and safety concerns of the derived adducts were paid less attention to. In this study, the optical isomers of di-MGO adducts of rutin, namely 6-(1-acetol)-8-(1-acetol)-rutin, were identified in foods and *in vivo*. After oral administration of rutin (100 mg/kg BW), these compounds reached the maximum level of 15.80 μg/L in plasma at 15 min, and decreased sharply under the quantitative level in 30 min. They were detected only in trace levels in kidney and fecal samples, while their corresponding oxidized adducts with dione structures presented as the predominant adducts in kidney, heart, and brain tissues, as well as in urine and feces. These results indicated that the unoxidized rutin-MGO adducts formed immediately after rutin ingestion might easily underwent oxidation, and finally deposited in tissues and excreted from the body in the oxidized forms. The formation of 6-(1-acetol)-8-(1-acetol)-rutin significantly mitigated the cytotoxicity of MGO against human gastric epithelial (GES-1), human colon carcinoma (Caco-2), and human umbilical vein endothelial (HUVEC) cells, which indicated that rutin has the potential to be applied as a safe and effective MGO scavenger and detoxifier, and AGEs inhibitor.

## Introduction

Methylglyoxal (MGO) is a typical reactive α-oxoaldehydes which can react very fast with proteins and nucleic acids to form advanced glycation end-products (AGEs) in the body. AGEs formation causes the dysfunction of functional proteins, ligates the receptor for advanced glycation end-products (RAGE), increases the production of reactive oxygen species (ROS), and triggers cellular apoptosis (1). Thus, the carbonyl stress caused by the excessive accumulation of MGO is always implicated in tissue damage in aging and various diseases, such as diabetic complications and cancer (2). Other than its reactions with body components *in vivo*, the appearance of MGO in foods also provides reactive precursors for the formation of AGEs, as well as the generation of potent carcinogens like acrylamide and methylimidazole (3, 4).

Nevertheless, MGO is ubiquitously present in all kinds of foods, especially in those containing high amounts of carbohydrates and lipids and underwent thermal processing. It is generated dominantly through the Maillard reaction in thermally processed foods when hexoses initially react with amino acids to generate Schiff bases, and convert to Amadori products, which undergo a serial of reaction steps to yield MGO (5). In foods containing high amounts of hexoses, such as honey, MGO is produced by the autoxidation of hexoses, which involves retro-aldol condensation, isomerisation and consequent fragmentation of sugars (6). In honey, another pathway to generate MGO is the dehydration of dihydroxyacetone (7). This is the main explanation of the high detection levels of MGO in the commercial Manuka honey since the precursor dihydroxyacetone present highly in this kind of honey (8). However, dihydroxyacetone do not contribute to the formation of MGO in other foods because of its absence. Oxidation of unsaturated fatty acids is another important pathway for the generation of MGO in fatty foods (5, 9). Moreover, MGO could also be generated through the microbial metabolism of dihydroxyacetone phosphate by the catalysis of methylglyoxal synthase, which explains its occurrence in some fermented beverages and foods (10). Therefore, given its ubiquitously appearance in foods, people are inevitably exposed to MGO through the consumption of foods. In fact, the deposit of MGO in human body is not only come exogenously from food ingestion, but also from the endogenous production through the glycolysis metabolism in animals (11). MGO is primarily produced in organisms by the degradation of triosephosphate intermediates during glycolysis (12). The plasma concentration of MGO in human samples varied from 96 to 652 nmol/L between different studies (13). It was further reported that MGO concentration elevated significantly under conditions of high glucose load (1). Scheijen and Schalkwijk reported a 31% increase in plasma concentration of MGO in patients with type 2 diabetes compared to the non-diabetic controls (13). Moreover, a maximum 6-fold elevation in MGO concentration could be reached in patients with renal disease (12).

Given the deleterious effects of MGO *in vivo* and the inevitable exposure of human being toward the exogenous and endogenous derived MGO, mitigation of MGO in both foods and *vivo* is highly demanded in these decades. As a result, various strategies have been investigated and applied to address this problem. They include the selection of raw materials, adjustment of processing parameters during food processing (14), and the addition of harmful substances inhibitors (15). Among these strategies, the use of natural compounds as MGO scavengers was considered the most applicable. They can be applied both for the control of hazardous compound generation in food, and disease protection or intervention *in vivo*.

Phenolic compounds are among the most applied natural compounds owing to their effective scavenging capacity of carbonyl compounds and other hazardous derivatives (16–18), in accompany with their distinguished physiological benefits (19–21). The flavonoids with typical *meta*-phenol structures possess efficient scavenging capacity for MGO. For example, epicatechin, (–)-epigallocatechin gallate and phloretin showed the highest elimination rate on MGO by 99% when incubated with MGO at 37°C for 24 h, and others like hesperetin, resveratrol and apigenin could also scavenge MGO by 63, 61, and 40%, respectively (16). Recent studies elucidated that these flavonoids eliminated MGO by means of reactions between the aromatic substitution and the electrophilic carbonyl group, which consequently brought various new products discovered (15, 17).

In our recent study, we found quercetin-3-*O*-rutinoside, usually called rutin, could eliminate 50% of MGO when incubated for 24 h under physiological conditions (22). Rutin is an important dietary flavonoid widely distributed in plant-derived food raw materials, such as vegetables, fruits, and grains (23). It has also been extensively used in food production and pharmaceutical development due to its health promoting properties, and the potential to prevent and treat various chronic diseases (24, 25). As a result, the Dietary Supplement Label Database have listed more than 860 commercial products containing rutin in the USA (24). This means humans are unavoidably exposed to rutin, and the reaction products of rutin and MGO formed both in food and *in vivo*.

However, although numerous flavonoid–MGO adducts have yet been reported as a consequent result of MGO elimination by flavonoids (15), few study focus on their generation levels in foods and *in vivo*, and much less attention has been paid to their metabolism and distribution *in vivo*, and the subsequent safety concerns. Some researchers found that myricetin (26), genistein (27, 28), and epicatechin (29) trapped MGO *in vivo* and produced mono- and di-MGO adducts. The formation levels of the flavonoid-MGO adducts in foods were, on the other hand, less investigated. We found that incorporation of quercetin and rutin-rich materials during food preparation yielded different adducts in the thermally processed foods (22, 30). Moreover, in contrast to the discovery of adducts in food and *in vivo*, the safety concerns of these neo-formed adducts were paid less attention and lack of evaluation, which might bring potential threaten to food safety and human health. In the last study (22), we identified three oxidized rutin-MGO adducts with novel dione structures in foods and *in vivo*, and found them to be less toxic than MGO in different cell lines. But in another study, we found that the unoxidized mono- and di-MGO adducts of quercetin formed during the preparation of biscuits displayed higher cytotoxicity than MGO in PC-12 cell line (30). This study aims to investigate the unoxidized rutin-MGO adducts formed between rutin and MGO, compare the differences in their generation and distribution in food and *in vivo* with those of the oxidized adducts, explain their metabolism *in vivo*, and evaluate their cytotoxicity against different cell lines related to their potential appearance *in vivo*.

## Materials and methods

### Materials and reagents

The commercial food products analyzed in this study were randomly purchased from the local market in Guangzhou, China. 40% aqueous solution of MGO was obtained from Macklin Biochemical Co., Ltd. (Shanghai, China). Standard compound of rutin was obtained from Ark Pharm, Inc. (IL, USA). Formic acid (HPLC grade) was obtained from Kermel Chemical Reagent Co., Ltd. (Tianjin, China). Methanol (HPLC grade) was purchased from Mallinckrodt Baker, Inc. (NJ, USA). CD_3_OD were purchased from Cambridge Isotope Laboratories, Inc. (MA, USA). Human gastric epithelial cell line (GES-1) and human colon carcinoma cell line (Caco-2) were bought from the American Type Culture Collection (ATCC, Rockville, MD, USA). Human umbilical vein endothelial (HUVEC) cell was bought from Shanghai iCell Bioscience Inc. (Shanghai, China). PBS was purchased from Boster Biological Technology Co., Ltd. (CA, USA). Trypsin–EDTA and MTT assay were obtained from Biosharp Co., Ltd. (Hefei, China). Fetal bovine serum (FBS), penicillin and streptomycin were purchased from Thermo Fisher Scientific Inc. (MA, USA).

### Reactions between rutin and MGO under simulated physiological condition

To investigate the unoxidized adducts formed between rutin and MGO, the simulated physiological reactions between rutin and MGO were established as described by Chen et al. (22). Briefly, 0.5 mM MGO and 0.5 mM rutin were reacted at 37°C for 24 h in PBS buffer (pH 7.4, 0.2 M). The reaction media were taken at 0.25, 0.5, 1, 2, 4, 8, 12, and 24 h, respectively for analyses. The formation of rutin–MGO adducts were detected at 360 nm by a high-performance liquid chromatography (HPLC) coupled with a diode array detector (DAD), and the yields were quantified through the external standard curves established with the standards prepared in Section 2.3 Preparation and purification of rutin–MGO adducts. The HPLC-DAD system consists of a Shimadzu LC-20AT HPLC system and an SPD-M20AVP diode array detector (Shimadzu Corporation, Kyoto, Japan). A Phenomenex Aeris Peptide XB-C18 column (4.60 mm × 250 mm, 5 μm; Torrance, CA) was applied for separation of rutin–MGO adducts. The chromatographic parameters and the eluting gradients were the same as described by Chen et al. (22).

### Preparation and purification of rutin–MGO adducts

In order to prepare enough unoxidized rutin–MGO adducts for latter the structural elucidation and quantification of adducts in foods and *in vivo*, the effects of pH, temperature, molar ratio, and reaction time on the formation of the adducts were investigated to obtain the optimal conditions for the preparation procedure. Firstly, the pH was set as 3, 5, 7.4, 9, and 11 when incubating 10 mM rutin with 50 mM MGO at 70°C for 9 h. Secondly, the temperature was set at 30, 50, and 70 °C when 10 mM rutin and 50 mM MGO were incubated at pH 7.4 for 9 h. Thirdly, the molar ratio of 1: 1, 1: 5, and 1: 10 were selected for rutin: MGO to be incubated at 70°C, pH 7.4 for 9 h, where the concentration of rutin was kept constantly at 10 mM. Finally, the reaction times of 4, 7, 10, 13, and 16 h was tested when 10 mM rutin reacted with 100 mM MGO at pH 7.4 under 70°C. At each point, the reaction solution was taken, filtered through 0.45 μm membrane, and analyzed by HPLC-DAD to calculate the yield of the adducts under different conditions. Finally, the standards of rutin-MGO adducts were prepared under the optimal conditions obtained.

The target adducts in the current study was hard to be separated by column chromatography applied in our previous study (22). Thus, these unoxidized adducts were separated using a LC-100 preparative medium-pressure liquid chromatograph equipped with a DAD (Shanghai Wufeng Scientific Instrument Co., Ltd., Shanghai, China). The column of Pntulips QC-C18 (10 mm × 250, 5 μm; Shanghai Puning Analysis Technology Co., Ltd., Shanghai, China) was applied for compound separation. A binary eluting gradient program was applied as follows using 0.1% aqueous formic acid as solvent A and methanol as solvent B: 0–8 min, 10–70% B; 8–11 min, 70–90% B; 11–13 min, 90% B; 13–14 min, 90–10% B; 14–22 min, 10% B. The flow rate was set at 3.0 mL/min, and the inject volume was 100 μL. The peaks of the adducts were monitored at 360 nm, and the corresponding adducts were collected. The adduct fractions with purity over ≥95% were combined, and the organic solvents were removed with a rotary evaporator. The pure adducts were then freeze-dried and stored at −20°C prior to NMR analysis and further analyses.

### Structural characterization of rutin–MGO adducts

To elucidate the structures of the rutin–MGO adducts formed, both HPLC-MS/MS analysis and nuclear magnetic resonance (NMR) analysis were conducted. The freeze-dried adduct was dissolved in methanol and analyzed with a Shimadzu Nexera X2 UHPLC coupled with a LCMS-8045 mass spectrometer (Shimadzu Corporation, Kyoto, Japan). The eluting solvents and the flow rate were the same as described in Section 2.2 Reactions between rutin and MGO under simulated physiological condition. The eluting program was modified as: 0–12 min, 10–70% B; 12–15 min, 70–90% B; 15–17 min, 90% B; 17–18 min, 90–10% B; 18–25 min, 10% B. The injection volume was 1 μL. The operating conditions of mass spectrometer were as follows: negative ion mode; scanning rage, *m/z* 100–1,000; pressure of drying gas and nebulizer gas, 50 psi; capillary voltage, 4 kV; declustering potential, 50 V; collision voltage, 25 eV; desolvation temperature, 300°C.

After the molecular structure were revealed by HPLC-MS/MS analysis, adducts A and B were further dissolved in CD_3_OD, and analyzed with a Bruker Avance III 600 MHz NMR spectrometer (Fällanden, Switzerland) to obtain ^1^H, ^13^C, HMQC, HMBC, and ROESY spectra for structure elucidation.

### Determination of rutin–MGO adducts in food samples

In the current study, multiple reaction monitoring (MRM) mode of HPLC-MS/MS analysis was applied to measure the formation levels of rutin–MGO adducts in 15 food samples brought randomly from the local market. The samples were prepared and analyzed as the same way as described by Chen et al. (22). The qualification and quantification fragmentation transitions for each adduct are as follows: Adduct A, *m/z* = 753 → 373 and 751 → 426 (collision energy, 48 eV and 43 eV, respectively) for qualification, and *m/z* = 753 → 401 (47 eV) for quantification; Adduct B, *m/z* = 753 → 426 and 751 → 444 (43 and 38 eV, respectively) for qualification, and *m/z* = 753 → 401 (48 eV) for quantification. The external calibration curve (*r* > 0.99) of each standard adduct prepared by our laboratory was used for quantification. The LOD (S/N > 3) and LOQ (S/N > 10) were 0.60 and 0.81 μg/L for adducts A, and 0.50 and 0.76 μg/L for adduct B, respectively.

### Determination of rutin–MGO adducts in plasma, urine, feces, and tissues of rats

The animals were treated as described in the study by Chen et al. (22). In brief, five 7 weeks old male Sprague–Dawley rats (190–220 g) were exposed to rutin (100 mg/kg BW) by oral gavage after fasting for 16 h. The plasma samples were collected at 0.25, 0.5, 1, 2, 4, 6, 8, 10, and 24 h after the administration, and stored under −80°C until analysis. The feces and urine were collected with the metabolic cages throughout the 24 h period. At 24 h, the rats were sacrificed after anesthetization, and the tissues including stomach, intestine (divided into duodenum, jejunum, ileum, caecum, and colon), heart, liver, kidney, and brain were taken and frozen immediately in liquid nitrogen and transferred to a deep freezer (−80°C) for storage before analysis. The procedures of the animal study followed the protocol by the Institutional Animal Care and Use Committee of the Laboratory Animal Center of Jinan University.

The plasma samples were treated as described by Chen et al. (22). About half gram of feces and tissue samples were weight accurately, and homogenized with 2 mL saline on an ice-bath. The homogenates were transferred to a centrifuge tube, added with 1 mL PBS (pH 5.0, 0.05 M) and 100 μL ascorbic acid (150 mg/mL), and extracted with 3 mL of methanol twice. The supernatants were combined after centrifugation at 13,000 × g under 4 °C for 10 min, evaporated to dryness, re-dissolved in 500 μL of 50% aqueous methanol containing 0.1% formic acid (v/v), and filtered through a 0.22 μm membrane for HPLC-MS/MS analysis. The adducts in 200 μL of urine were extracted twice with 1 mL methanol. The supernatants were collected after centrifugation at 13,000 × g under 4°C for 10 min, and evaporated to dryness. The residues were dissolved in 200 μL of 50% aqueous methanol containing 0.1% formic acid (v/v), and filtered through a 0.22 μm membrane for HPLC-MS/MS analysis. A sample of 1 μL was injected into an Agilent Technologies 1,260 Infinity HPLC (Agilent Technologies, CA, USA) interfaced to an AB 4500 Q-Trap mass spectrometer (AB Sciex, MA, USA) in the negative mode for quantification of adducts A and B, respectively. The eluting programs for HPLC analysis, and the operation conditions of mass spectrometer refers to those established by Chen et al. (22). The qualification and quantification fragmentation transitions for each adduct are the same as provided in Section 2.5 Determination of rutin–MGO adducts in food samples. Moreover, as the information on the occurrence levels of 6-(1,2-propanedione)-8-(1-acetol)-rutin, 6-(1-acetol)-8-(1,2-propanedione)-rutin, and 6-(1,2-propanedione)-8-(1,2-propanedione)-rutin discovered in the last study was still missing, the concentration of these oxidized rutin-MGO adducts in various tissues and urine and feces were also determined in this study. The qualification and quantification fragmentation transitions of these adducts refers to those provided previously (22).

### Cytotoxicity evaluation of rutin–MGO adducts in four different cell lines

The cytotoxicity of the unoxidized rutin–MGO adducts were tested against the GES-1, Caco-2, and HUVEC cell lines representing gastrointestinal and circulation systems. The cells were cultivated as described by Chen et al. (22), and treated with 0, 200, 400, 600, 800, 1,000 μM of rutin, MGO, adduct A, and adduct B for 24 h, after which the viability of the cells was evaluated by MTT assay (22).

### Statistical analysis

All the experiments were performed in triplicate, and the results were expressed as mean ± standard deviation. Statistical analyses were conducted with SPSS Statistics 25.0 (SPSS, Inc., Chicago, IL, USA). One-way analysis of variance (ANOVA) was applied to investigate the differences between samples or treatments (*P* < 0.05). Furthermore, multiple comparisons between different samples were carry out by the Duncan's test at significant level of 0.05.

## Results and discussions

### Formation of rutin-MGO adducts under simulated physiological condition

In our previous study, we found that rutin displayed considerable scavenging capacity for MGO under simulated physiological condition. 50% of MGO was eliminated when rutin (0.5 mM) was incubated with MGO (0.5 mM) under 37 °C for 24 h (22). Moreover, three major adducts were detected as a consequence of the elimination reaction. They were unambiguously identified to be 6-(1,2-propanedione)-8-(1-acetol)-rutin (C_33_H_36_O_20_), 6-(1-acetol)-8-(1,2-propanedione)-rutin (C_33_H_36_O_20_), and 6-(1,2-propanedione)-8-(1,2-propanedione)-rutin (C_33_H_34_O_20_), all of which were oxidized adducts of rutin and MGO with dione structures. This was the first report of polyphenol-MGO adducts with oxidized dione structures. However, the majority of investigations on the scavenging mechanism of polyphenols on MGO reported the formation of unoxidized adducts between polyphenols and MGO. Yoon and Shim (31) reported that non-oxidized rutin–di-MGO adducts were formed when incubating rutin and MGO for 24 h, and speculated it contributed to the highly inhibitory effects (more than 90%) of rutin on the formation of AGEs in glycation reaction (31).

**Figure 1:**
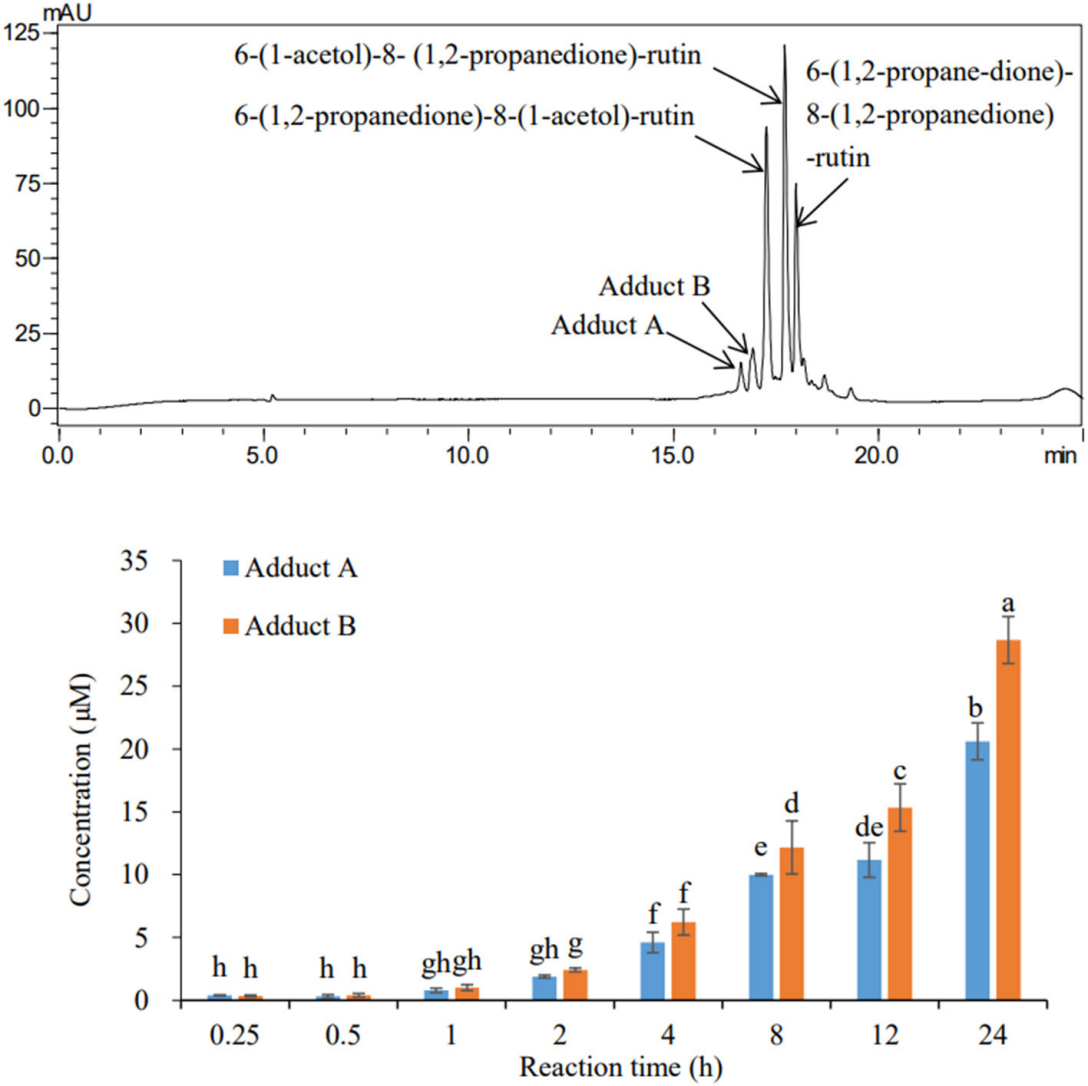
HPLC chromatogram (upper) of the adducts and their formation levels (bottom) during 24 h incubation of rutin and MGO under simulated physiological condition.

From the HPLC chromatogram obtained after incubation of rutin and MGO under simulated physiological condition (Figure 1), two peaks, hereby named adduct A and B, were also formed at detectable levels other than the three oxidized adducts with dione structures reported (22). The formation of these two adducts also showed a time-dependent manner. Through the quantification of adducts with external standard curves obtained by the adduct standards prepared in the next sections, the yields of these adducts at different periods under the simulated physiological condition were determined. At the first 15 min, the contents of adducts A and B were only 0.74 μM in total, accounting for 6% of the total adducts (the sum of three oxidized adducts and adducts A and B). As the incubation time prolonged, the yield of the adducts hardly increased within 1 h, but started to increase significantly after 4 h and reached finally a total of 49.27 μM at 24 h. Adducts A and B were generated at levels of 20.61 and 28.67 μM, respectively, at the reaction time of 24 h. While by that time, the adducts 6-(1,2-propanedione)-8-(1-acetol)-rutin, 6-(1-acetol)-8-(1,2-propanedione)-rutin, and 6-(1,2-propanedione)-8-(1,2-propanedione)-rutin identified in the last study (22) were produced at levels of 20.49, 21.18, and 53.35 μM, respectively.

### Preparation and structural elucidation of rutin-MGO adducts

**Figure 2:**
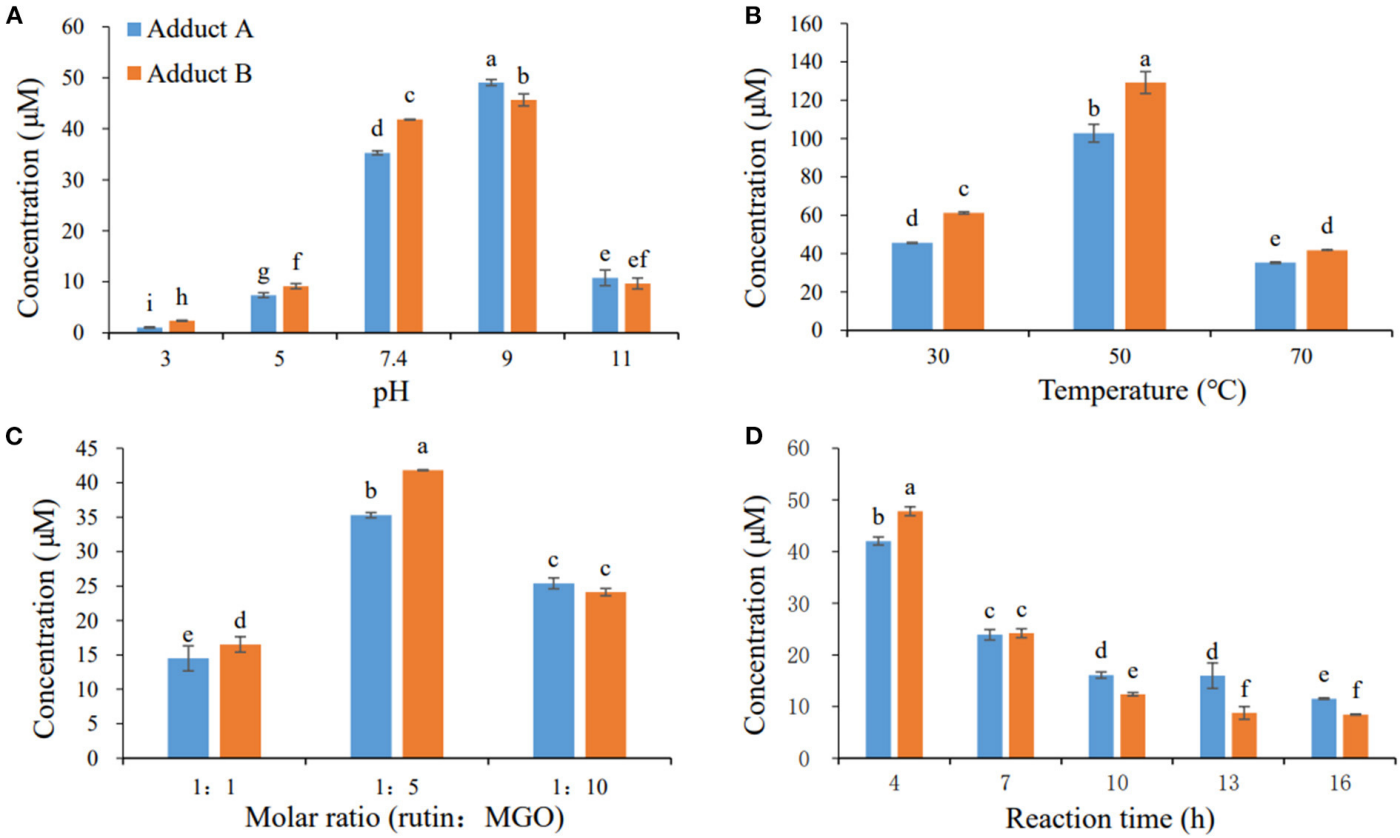
Effects of pH **(A)**, temperature **(B)**, molar ratio **(C)**, and reaction time **(D)** on the formation of adducts A and B. Different letters represent significant differences (*P* < 0.05).

In our previous study, adducts A and B could not be successfully separated and purified by the column chromatograph with all kinds of absorbents tried (22). Thus, the preparative medium-pressure liquid chromatograph was applied to obtain the standard adducts. Firstly, the effects of different reaction parameters on the yield of these two adducts were investigated to optimize the preparation conditions for the adducts. As shown in Figure 2A, the formation of adducts A and B increased as the pH of the medium elevated from 3 to 9, and decreased dramatically when the pH further increased. The yields of these two adducts increased as the reaction temperature increased from 30 to 50°C, and decreased when the temperature further elevated to 70°C (Figure 2B). The adducts reached maximum yields at the substrate molar ratio of 1:5 for rutin:MGO, while higher or lower substrate molar ratio all decreased the formation of these two adducts (Figure 2C). Figure 2D showed that the formation of both adducts A and B decreased as the reaction time prolonged. In contrast, a highly oxidized adduct of rutin and MGO, 6-(1,2-propanedione)-8-(1,2-propanedione)-rutin, was formed moderately (42.53 μM compared to 42.04 and 47.81 μM for adducts A and B, respectively) under the same condition at 4 h, and increased dramatically as the reaction proceeded until 16 h, which reached maximum of 122.20 μM. While at 16 h, the yields of adducts A and B decreased to minimum of 11.53 and 8.42 μM, respectively. As the adducts A and B were latter identified to be unoxidized di-MGO adducts of rutin, this indicated that the unoxidized adducts might undergo oxidation reactions and be predominantly converted to the highly oxidized products as the reaction proceeded. As a result, the optimal reaction condition chosen for the preparation of the target adducts A and B was as follows: 10 mM rutin and 50 mM MGO were combined and reacted at 50 °C and pH 9 for 4 h. When the reaction ends, the reactants were separated by preparative medium-pressure liquid chromatograph to obtain the highly purified (≥ 95%) standards of the adducts for structural elucidation.

The mass spectra displayed in Table 1 showed that adducts A and B were isomers with the same molecular ion [M-H]^−^ detected at *m/z* 753 and fragment ions at *m/z* 444, 425 and 401. The *m/z* 753 [M-H]^−^ is 144 mass units greater than the *m/z* 609 [M-H]^−^ of rutin. Thus, they were preliminarily identified as conjugates of the rutin molecule with two moieties of MGO attached, whose molecular formula were both C_33_H_38_O_20_. Abundant investigations pointed the trapping of α-dicarbonyl compounds dominantly happened at C-6 and C-8 positions of the flavonoids with *meta*-phenol structures. Yoon and Shim (31) detected previously a di-MGO-rutin adduct after incubation of rutin and MGO for 24 h. However, only mass spectrometric analysis was conducted for the structural elucidation, which was hard to provide unambiguous information on the adduct structure (31). Therefore, we isolated the two adducts by the preparative medium-pressure liquid chromatograph for NMR analysis. The ^1^H and ^13^C NMR data were listed in Table 1, and the spectra of heteronuclear multiple bond correlation (HMBC), heteronuclear multiple quantum correlation (HMQC) and Rotating Frame Nuclear Overhauser Effect Spectroscopy (ROESY) analyses were provided in Supplementary Figures S1, S2, for adducts A and B, respectively. According to all the NMR data obtained, adducts A and B were assigned to be both as 6-(1-acetol)-8-(1-acetol)-rutin, in which MGO attacked the *ortho*-position at C-6 and C-8 in the aromatic A ring. The chemical shifts in adducts A and B were very close to each other, the main difference between the two compounds in the NMR spectra is that the chemical shifts of the corresponding carbons at C-11 and C-14 are more than 1.10 ppm, and the corresponding chemical shifts of hydrogen are more than 0.10 ppm, indicating that C-11 and C-14 in the two compounds possess different chiral centers. Therefore, adducts A and B were supposed to be optical isomers, which possess identical planar molecular structures.

**Table 1:** Structure, mass spectra, ^1^H (600 MHz), and ^13^C (126 MHz) NMR data of Adduct A and B.

	** 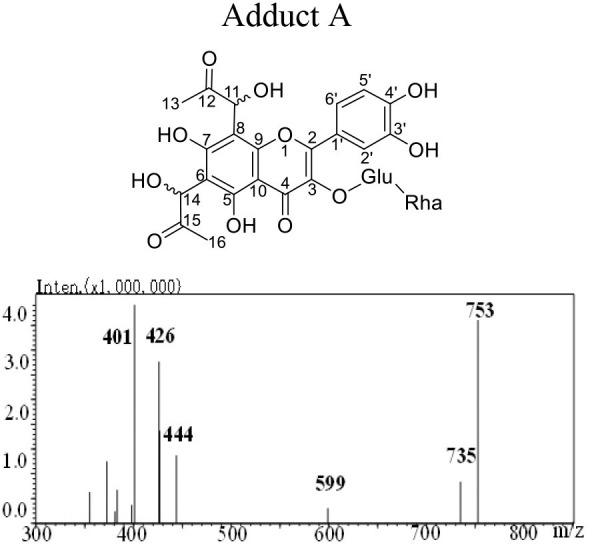 **	** 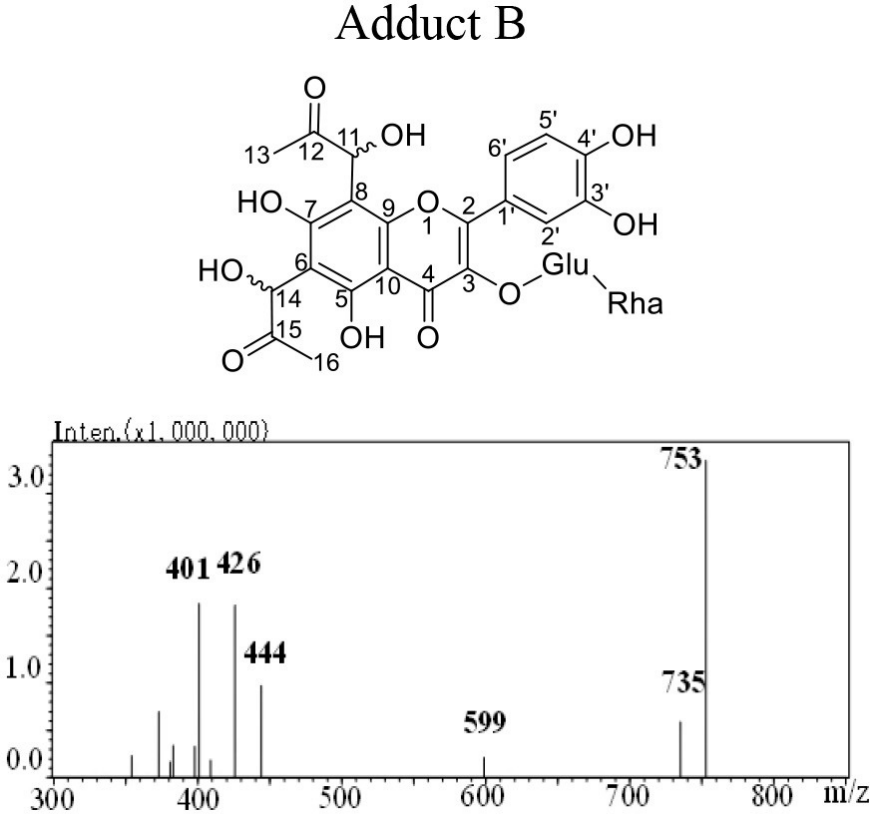 **		
**No**.	**δ_H_ (ppm)**	**δ_C_ (ppm)**	**δ_H_ (ppm)**	**δ_C_ (ppm)**
2	-	146.08	-	146.07
3	-	135.47	-	135.43
4	-	179.98	-	179.85
5-OH	-	165.81	-	165.15
6	-	103.97	-	103.81
7-OH	-	162.86	-	162.83
8	-	103.54	-	103.49
9	-	159.50	-	159.38
10	-	102.49	-	102.51
11	5.58 (s, 1H)	70.23	5.47 (s, 1H)	71.83
12	-	209.32		209.68
13	2.15 (s, 3H)	25.93	2.17 (s, 3H)	25.81
14	5.44 (s, 1H)	68.66	5.34 (s, 1H)	69.72
15	-	209.32		209.16
16	1.71 (s, 3H)	23.13	1.69 (s, 3H)	21.41
1'	-	123.80	-	123.85
2'	7.69 (s, 1H)	116.11	7.63 (s, 1H)	116.10
3'-OH	-	145.93	-	145.94
4'-OH	-	150.05	-	150.06
5'	6.90 (d, *J* = 8.5 Hz, 1H)	117.69	6.88 (d, *J* = 8.5 Hz, 1H)	117.68
6'	7.69 (d, *J* = 8.5 Hz, 1H)	122.78	7.63 (d, *J* = 8.5 Hz, 1H)	122.80
***3-O-glu***				
1”	5.23 (d, *J* = 7.8 Hz, 1H)	107.23	5.27 (d, *J* = 7.8 Hz, 1H)	106.32
2”	3.16-3.82 (m, 10H, H-2”–H-6”, H-2”'−5”')	75.66	3.18-3.87 (m, 10H, H-2”–H-6”, H-2”'−5”')	75.66
3”		78.11		78.10
4”		71.77		72.00
5”		77.28		77.37
6”		68.91		68.79
***6*** **”** ***-O-rha***				
1”'	4.49 (s, 1H)	108.77	4.48 (s, 1H)	107.17
2”'	3.16–3.82 (m, 10H, H-2”–H-6”, H-2”'−5”')	72.05	3.18–3.87 (m, 10H, H-2”–H-6”, H-2”'−5”')	72.05
3”'		72.19		72.19
4”'		73.86		73.85
5”'		69.68		69.68
6”'	1.13 (d, *J* = 6.2 Hz, 3H)	17.90	1.12 (d, *J* = 6.0 Hz, 3H)	17.91

### Determination of 6-(1-acetol)-8-(1-acetol)-rutin in commercial foods

Flavonoids could efficiently trap MGO generated during thermal processing of carbonhydrate and lipid rich food materials by the formation of corresponding adducts (15). Rutin is widely distributed in various grains. Its content was announced to be the highest in buckwheat, which ranges from 0.17 to 17.95 g/kg (32). Other grains, such as wheat, barley, quinoa, oat, and potato also contained rutin in moderate levels up to 38.3 mg/kg (33), 20.6 mg/kg (34), 5.8 mg/kg (35), 3.2 mg/kg (36), and 36 mg/kg (37), respectively. Therefore, the occurrence of rutin-MGO adducts in thermally processed foods were expected. To determine whether the unoxidized adducts of rutin and MGO exist in foods, 15 different food products randomly collected from the local market were analyzed. However, only one sample (code 4 listed in Table 2) contained detectable amount of unoxidized rutin-MGO adducts, with the values of 0.27 mg/kg and 0.89 mg/kg for adducts A and B, respectively. The contents of the oxidized adducts were also determined, which were 0.41 ± 0.07, 2.85 ±0.17, and 0.29 ± 0.09 mg/kg, respectively, for 6-(1,2-propanedione)-8-(1-acetol)-rutin, 6-(1-acetol)-8-(1,2-propanedione)-rutin, and 6-(1,2-propanedione)-8-(1,2-propanedione)-rutin. The sum of oxidized adducts were 3-folds higher than that of the unoxidized ones. Furthermore, samples with codes of 1, 3, and 5-9 were investigated in our previous study (22) and also showed considerable amount of oxidized adducts in ranges of 0.15–1.43, 0–2.43, and 0.29–2.79 mg/kg for 6-(1,2-propanedione)-8-(1-acetol)-rutin, 6-(1-acetol)-8-(1,2-propanedione)-rutin, and 6-(1,2-propanedione)-8-(1,2-propanedione)-rutin, respectively. All of these might indicate that the unoxidized rutin-MGO adducts would undergo oxidation during processing and storage of the foods after their formation, and predominantly exist in oxidized forms in the commercial products.

**Table 2:** Contents (mg/kg) of adducts A and B in commercial food samples.

**Code**	**Product**	**Grain ingredients**	**Adduct A**	**Adduct B**
1	Coarse multi-grain biscuit 1	Oat flour, barley flour, buckwheat flour, quinoa flour	N.D.^a^	N.D.
2	Coarse multi-grain biscuit 2	Coarse rice flour, tapioca starch	N.D.	N.D.
3	Multi-grain bar 1	Buckwheat, wheat, barley, oat	N.D.	N.D.
4	Multi-grain bar 2	Buckwheat, wheat, barley, oat	0.27 ± 0.02	0.89 ± 0.05
5	Oat biscuit	Wheat, oat	N.D.	N.D.
6	Biscuit	Wheat	N.D.	N.D.
7	Cracker	Wheat	N.D.	N.D.
8	Potato chip 1	Potato	N.D.	N.D.
9	Potato chip 2	Potato	N.D.	N.D.
10	Shortbread	Wheat flour, seasame	N.D.	N.D.
11	Quinoa cracker	Wheat flour, oat, seasame, quinoa	N.D.	N.D.
12	Quinoa rolls	Wheat flour, quinoa flour	N.D.	N.D.
13	Nut bar	Rice, coarse rice, cashew, almond	N.D.	N.D.
14	Coarse grain cracker	Corn, black rice	N.D.	N.D.
15	Buckwheat noodle	Rye flour, buckwheat flour	N.D.	N.D.

^a^N.D., not detected.

### Plasma concentration variation of 6-(1-acetol)-8-(1-acetol)-rutin after rutin administration

To satisfy the need of pharmacokinetics investigation of exogenous substances such as those in foods and drugs, various methods have been developed and applied including radiography, fluorecent analysis, liquid chromatography, and LC-MS/MS. Among them, LC-MS/MS analysis was the most promised with much better specificity, less limitation and wider application range (38, 39). MRM mode of HPLC-MS/MS analysis has shown advantages for the identification and determination of exogenous substances and their metabolites in complex biological samples especially when the compounds were at extremely low-abundance levels (39, 40), and therefore, was applied herein to measure the concentration levels of rutin–MGO adducts in plasma and organs to reveal the formation, metabolism and distribution of adducts *in vivo*. As shown in Table 3, 9.40 and 6.40 μg/L of adducts A and B, respectively, were detected in the plasma 15 min after the administration of rutin. At that time, the formation of oxidized rutin-MGO adducts, 6-(1,2-propanedione)-8-(1-acetol)-rutin, 6-(1-acetol)-8-(1,2-propanedione)-rutin, and 6-(1,2-propanedione)-8-(1,2-propanedione)-rutin were 2.38, 1.61, and 1.02 μg/L, respectively (22). These results indicated that rutin reacts with endogenous MGO after ingestion, and form mainly unoxidized adducts initially. But these unoxidized adducts remained in the plasma only for a very short period, with none or only trace levels of these adducts detected after 30 min of rutin administration. Compared to the sharp concentration decline of unoxidized adducts, the oxidized adducts decreased gradually to 0.39–0.67 μg/L after 4 h, and remained constantly at these levels until 24 h. This indicated that the unoxidized adducts are easily metabolized or excreted through circulation, whereas the oxidized ones are much more stable and retained longer in blood circulation. In an anesthetized, mesenteric lymphatic/duodenum-cannulated rat model, the plasma concentration of rutin reached maximum at 60 min, and declined afterwards (41). Therefore, the structural differences of these compounds should determine to a large extent to their metabolic and pharmacokinetic properties *in vivo*.

**Table 3:** Plasma concentration of adducts A and B in rats administrated with 100 mg/kg BW rutin.

**Time point (h)**	**Adduct A (μg/L)**	**Adduct B (μg/L)**
0.00	N.D.^a^	N.D.
0.25	9.40 ± 6.37	6.40 ± 3.90
0.50	N.D.	N.D.
1.00	trace	trace
2.00	trace	trace
4.00	N.D.	N.D.
6.00	N.D.	N.D.
8.00	N.D.	trace
10.00	trace	N.D.
24.00	N.D.	trace

^a^N.D., not detected.

### Distribution of different rutin-MGO adducts in tissues of rats

Furthermore, we investigated all of the unoxidized and oxidized adducts formed between rutin and MGO after 24 h of rutin administration. This was not conducted in the previous study (22). We found that the unoxidized adducts A and B were hardly remained in all the tissues after 24 h. Only trace amount of unoxidized adducts were detected in the kidney. The highly oxidized adduct, 6-(1,2-propanedione)-8-(1,2-propanedione)-rutin, was detected at the level of 0.10 μg/kg in stomach, but was not detected over LOQ level in the intestine. The two moderately oxidized adducts, 6-(1,2-propanedione)-8-(1-acetol)-rutin and 6-(1-acetol)-8-(1,2-propanedione)-rutin, were hardly detected at quantitative levels in the gastrointestinal tract. Interestingly, although these oxidized adducts hardly existed in the digestive tract, their occurrence in organs of kidney, heart and brain were detected. In liver, only 6-(1,2-propanedione)-8-(1,2-propanedione)-rutin existed in dosable level (0.09 μg/kg). These data indicated that the unoxidized rutin-MGO adducts, which displayed higher pharmacokinetic rate, could hardly accumulate in tissues. While the oxidized rutin-MGO adducts were the predominant deposit forms of the adducts between rutin and MGO, and lasting in tissues for long periods after oral administration. Rutin has been reported to possess various biological activities and present clinically relevant functions, but is of poor bioavailability which is mainly owing to its low solubility. Studies indicated that changes of the solubility of rutin, not only in the aqueous phase but also in lipid phase, might influence greatly the bioavailability of rutin (24). For example, the rutin concentration in brain of rats intranasally administrated with rutin loaded chitosan nanoparticles was significantly 7-folds higher than that in rats treated with rutin solution (42). Thus, it was speculated that the oxidized structures of rutin-MGO adducts might change the solubility properties and the bioavailability of the compounds, and facilitate them to pass the blood-brain barrier in rats.

### Excretion of different rutin-MGO adducts *via* urine and feces

In the current study, the urine and fecal samples were collected within 24 h after the rats were orally administrated with rutin (100 mg/kg BW). Different unoxidized and oxidized adducts were detected and quantified with the HLPC-MS/MS method developed and the corresponding synthetic adduct prepared by our laboratory as the authentic standard. As shown in Table 4, the unoxidized adducts were not detected in any urine samples. They appeared in the feces only at detectable levels. In contrast, the three oxidized adducts was all detected at quantitative levels in feces. Moreover, the adducts oxidized at one moiety of MGO substitute were the major forms of the adducts excreted in feces. The content of the highly oxidized 6-(1,2-propanedione)-8-(1,2-propanedione)-rutin was 6–9-folds <6-(1,2-propanedione)-8-(1-acetol)-rutin and 6-(1-acetol)-8-(1,2-propanedione)-rutin. In urine samples, only 6-(1-acetol)-8-(1,2-propanedione)-rutin and 6-(1,2-propanedione)-8-(1,2-propanedione)-rutin were detected at levels of 0.09 and 0.10 μg/L, respectively. The other adducts were not found or only observed in tract amount over the LOD level. The excretion of unoxidized flavonoid-MGO adducts from urine and feces has been reported by other researchers in acute and chronic studies of mice. Zhang et al. treated mice with 100–400 mg/kg BW of myricetin by oral gavage, and detected two mono-MGO-myricetin adducts in the urine and fecal samples collected afterwards (26). Huang et al. observed there were three isomers of mono-MGO adducts of epicatechin and seven isomers of di-MGO adducts in urine samples collected from the mice fed with 200 mg/kg BW of epicatechin and 1.0 g/kg BW of MGO (29). In contrast to our findings, Wang et al. (27) observed both the mono- and di-MGO adducts of genistein in the *in vitro* study, but only found two peaks corresponding to the mono-MGO adducts *in vivo* by selected ion monitoring (SIM) in HPLC-MS analysis. This indicated that different flavonoid structures would derive totally different adducts *in vivo* after trapping of MGO. Moreover, this was the first time that the oxidized flavonoid-MGO adducts were found *in vivo*, and displayed to be the predominant forms excreted and accumulated in certain tissues, which might indicate that the unoxidized flavonoid-MGO adducts would undergo further oxidation to yield oxidized adducts during digestion and metabolism.

**Table 4:** Concentrations of rutin-MGO adducts in tissues, urine, and feces of rats after 24 h of rutin administration (100 mg/kg BW).

**Tissue**	**Concentration (**μ**g/kg for tissues and feces;** μ**g/L for urine)**				
	**6-(1,2-propanedione)-8-(1-acetol)-rutin**	**6-(1-acetol)-8-(1,2-propanedione)-rutin**	**6-(1,2-propanedione)-8-(1,2-propanedione)-rutin**	**Adduct A**	**Adduct B**
Stomach	N.D.^a^	trace	0.10 ± 0.01	N.D.	N.D.
Duodenum	N.D.	N.D.	N.D.	N.D.	N.D.
Jejunum	N.D.	N.D.	N.D.	N.D.	N.D.
Ileum	trace	trace	trace	N.D.	N.D.
Caecum	N.D.	N.D.	trace	N.D.	N.D.
Colon	N.D.	trace	N.D.	N.D.	N.D.
Liver	trace	trace	0.09 ± 0.01	N.D.	N.D.
Kidney	0.07 ± 0.01	0.12 ± 0.02	0.16 ± 0.04	trace	trace
Heart	0.09 ± 0.03	trace	0.14 ± 0.09	N.D.	N.D.
Brain	0.11 ± 0.02	0.45 ± 0.28	0.12 ± 0.02	N.D.	N.D.
Urine	trace	0.09 ± 0.00	0.10 ± 0.01	N.D.	N.D.
Feces	1.54 ± 0.49	2.23 ± 0.54	0.26 ± 0.18	Trace	Trace

^a^N.D., not detected.

### Cytotoxicity of 6-(1-acetol)-8-(1-acetol)-rutin in different cell lines

**Figure 3:**
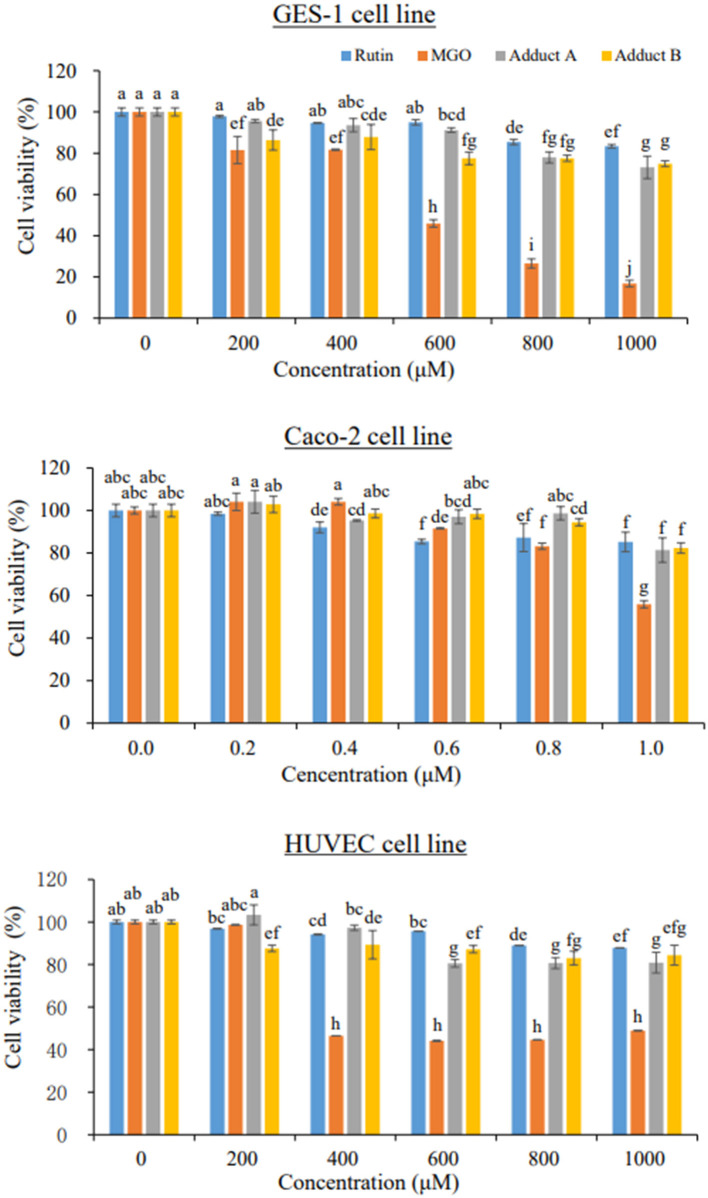
Cell viability of GES-1 (upper), Caco-2 (middle), and HUVEC cells (bottom) under 24 h treatments by different concentrations of rutin, MGO, adduct A, and adduct B. Different letters represent significant differences (*P* < 0.05).

Although the flavonoid-MGO adducts were proven to be formed both in foods and *in vivo* in the current and the other studies (22, 26–30), their safety concerns were hardly evaluated. Gastric and intestinal mucosae form the barrier between the body and the ingested substances in the lumens (43). Endothelial cells are involved in the exchanges of metabolites between blood and tissues (44). Thus, both the gastrointestinal epithelial cells and the endothelial cells could be target of numerous xenobiotics. Given the discovery of unoxidized rutin-MGO adducts in both the commercial foods and in animal plasma, evaluation of the toxicity of these adducts are very important for the sake of food safety concerns. Cell proliferation was thought to be the primary point of control in the regulation of normal tissue kinetic homeostasis, and has been the major focus of the etiology of diseases (45). Therefore, in order to get a first insight and assessment on the safety of the formation of flavonoid-MGO adducts, we evaluated the cytotoxicity of the rutin-MGO adducts toward the GES-1, Caco-2 and HUVEC cell lines representing gastrointestinal epithelial cells and vascular endothelial cells by determination of the cell viability under different treatment levels of the adducts in comparison to those of MGO and rutin (43, 44, 46). As shown in Figure 3, MGO displayed remarkable cytotoxicity toward GES-1 in a clear dose-dependent manner. The growth of the cells was inhibited by 18–19% when treated with 200–400 μM MGO, while more than 50% was inhibited when treated with 600 μM MGO. When the treatment level of MGO further increased to 1 mM, only 17% of the cells left alive. In comparison, the viability of HUVEC cells was not impacted by 200 μM MGO treatment, while decreased significantly to 47% when treated with 400 μM MGO, then became resistant and kept within the range of 44–49% at treatment levels of 600–1,000 μM. The Caco-2 cells were more resistant toward MGO treatment than both GES-1 and HUVEC cells as observed previously in our other studies (22, 47). The viability of Caco-2 cells was between 83 and 100% when treated with 200–800 μM of MGO. It decreased further to 56% when treated with 1.0 mM MGO. In comparison to MGO, rutin displayed much less toxicity toward all the cells tested. Only 16, 15, and 12% of the cells were dead under the highest treatment level (1 mM) of rutin in GES-1, Caco-2, and HUVEC cell lines, respectively. Adducts A and B also displayed comparable low cytotoxicity toward the three cell lines. As shown in Figure 3, treatment with 1 mM of adducts A and B reduced the cell viability by 27 and 25% in GES-1 cell line; by 19 and 18% in Caco-2 cell line; and by 19 and 16% in HUVEC cell line, respectively. Through the comparison of cell proliferation under different treatments with MGO, rutin and the adducts, it was indicated that the formation of unoxidized rutin-MGO adducts significantly mitigated the cytotoxicity of MGO to a level comparable to that of rutin. Since the oxidized adducts formed between rutin and MGO also displayed much lower toxicity toward these gastrointestinal epithelial cell lines and vascular endothelial cell line compared to that of MGO (22), the scavenging of MGO by rutin and the consequent formation of various rutin-MGO adducts were suggested not to only inhibit the formation of AGEs, but also to lower the toxicity of MGO.

## Conclusion

In our previous study, we identified for the first time three oxidized rutin-MGO adducts with dione structures formed in foods and *in vivo*. This study focused on the unoxidized rutin-MGO adducts. Through separation and purification, the di-MGO adducts of rutin obtained were identified by HPLC-MS/MS and NMR analysis to be optical isomers of 6-(1-acetol)-8-(1-acetol)-rutin. They were detected in commercial thermal processed foods, although in much less amounts than the oxidized rutin-MGO adducts detected previously (22). The animal study showed that 6-(1-acetol)-8-(1-acetol)-rutin were first formed in larger amount than the oxidized rutin-MGO *in vivo*, and appeared in the blood circulation at a total level of 15.80 μg/L after 15 min of oral gavage of rutin. But they were detected only in trace levels in the blood circulation after 30 min of rutin administration, which indicated that they might be quickly metabolized (including oxidation to oxidized adducts) or excreted. In the 24 h urine and fecal samples collected, the oxidized adducts again presented as the predominant adducts excreted. Moreover, the unoxidized rutin-MGO adducts were hardly detected in all the tissues investigated, while the oxidized adducts still deposited in detectable amount in kidney, heart and brain after 24 h of oral intake of rutin. Considering their appearance in foods and *in vivo*, the cytotoxicity of the adducts were evaluated in gastrointestinal epithelial cells and vascular endothelial cells, and were proven to be much lower than their precursors of MGO. The results of this study further promised the application of rutin as an effective MGO scavenger and AGEs inhibitor both in food production and for health intervention.

## Data availability statement

The original contributions presented in the study are included in the article/Supplementary material, further inquiries can be directed to the corresponding author/s.

## Ethics statement

The animal study was reviewed and approved by Institutional Animal Care and Use Committee of the Laboratory Animal Center of Jinan University.

## Author contributions

Conceptualization: JZ, WZ, and YX. Methodology: JZ, CH, and JO. Data curation, funding acquisition, and supervision: JZ. Investigation: MC, HZ, YX, and PL. Formal analysis: JH and JO. Validation: JH and YX. Writing—original draft: PL and JZ. Writing—review & editing: JZ, HZ, CH, and HE-N. Project administration: JZ and WZ. All authors contributed to the article and approved the submitted version.

## Funding

This work was financed by the National Natural Science Foundation of China (grant number 31972180); the Basic and Applied Basic Research Fund of Guangdong Province (grant numbers 2019A1515011967, 2021A1515010822, and 2019A1515011994); the Fundamental Research Funds for the Central Universities of Jinan University (grant number 21620107); the Pearl River Talent Program (grant number 20170096) by Guangdong Science and Technology Department, Guangdong, China.

## Conflict of interest

Authors WZ and YX were employed by the company “Dongguan Silang Foods Co., Ltd.”

The remaining authors declare that the research was conducted in the absence of any commercial or financial relationships that could be construed as a potential conflict of interest.

## Publisher's note

All claims expressed in this article are solely those of the authors and do not necessarily represent those of their affiliated organizations, or those of the publisher, the editors and the reviewers. Any product that may be evaluated in this article, or claim that may be made by its manufacturer, is not guaranteed or endorsed by the publisher.
