# Supplementary material for: Formation and metabolism of 6-(1-acetol)-8-(1-acetol)-rutin in foods and in vivo, and their cytotoxicity
Source: Front Nutr. 2022 Aug 2;9:973048. doi: 10.3389/fnut.2022.973048 (PMC9378861; doi:10.3389/fnut.2022.973048)
Supplement: Supplementary file 1 [file Data_Sheet_1.docx]

Supplementary Material

## Supplementary Figures

A

B


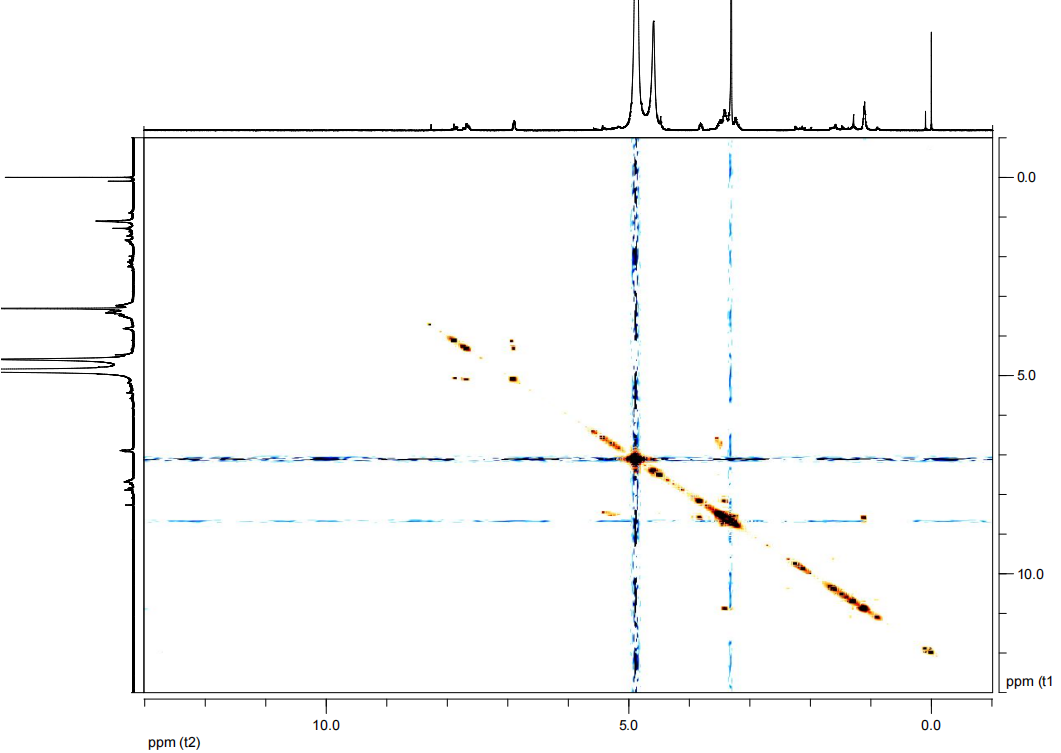


C

**Supplementary Figure 1.** HMQC (A), HMBC (B), and ROESY (C) spectra of adduct A.

A

B


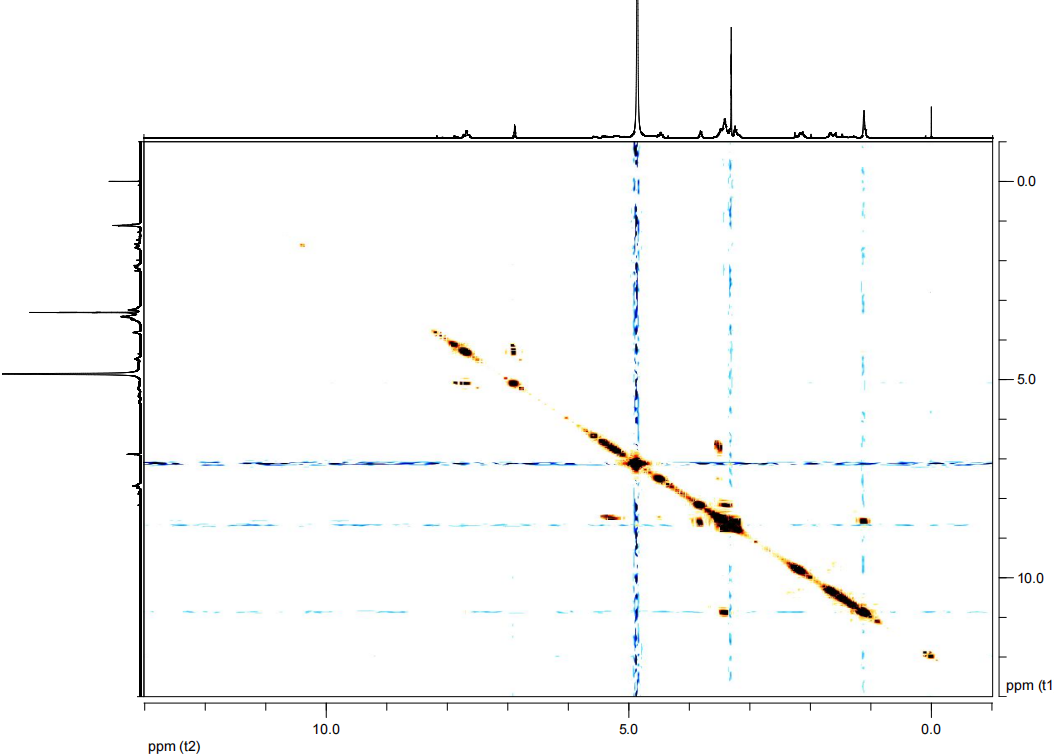


C

**Supplementary Figure 2.** HMQC (A), HMBC (B), and ROESY (C) spectra of adduct B.
